# Fragile X Syndrome: Prevalence, Treatment, and Prevention in China

**DOI:** 10.3389/fneur.2017.00254

**Published:** 2017-06-06

**Authors:** Manman Niu, Ying Han, Angel Belle C. Dy, Junbao Du, Hongfang Jin, Jiong Qin, Jing Zhang, Qinrui Li, Randi J. Hagerman

**Affiliations:** ^1^Department of Pediatrics, Peking University First Hospital, Beijing, China; ^2^MIND Institute, University of California-Davis Medical Center, Sacramento, CA, United States; ^3^Ateneo de Manila University – School of Medicine and Public Health, Quezon, Philippines; ^4^Department of Pediatrics, Peking University People’s Hospital, Beijing, China; ^5^Department of Pediatrics, University of California-Davis Medical Center, Sacramento, CA, United States

**Keywords:** fragile X syndrome, prevalence, treatment, prevention, China

## Abstract

Fragile X syndrome (FXS) is the most common inherited cause of intellectual disability and the leading monogenic cause of autism spectrum disorder. Although FXS has been studied for several decades, there is relatively little basic science or clinical research being performed on FXS in China. Indeed, there is a large gap between China and Western countries in the FXS field. China has a potentially large number of FXS patients. However, many of them are underdiagnosed or even misdiagnosed, and treatments are not always administered in the Chinese population. This review discusses the prevalence, treatment, and prevention of FXS in China to facilitate an understanding of this disease in the Chinese population.

## Introduction

Fragile X syndrome (FXS) is the most significant monogenic cause of intellectual disability (ID) and autism spectrum disorder (ASD). FXS is caused by an expansion of CGG repeats >200 in the 5′ untranslated region of the fragile X mental retardation 1 gene (*FMR1*), which is located on *Xq27.3*. The abnormal CGG expansion leads to methylation and transcriptional silencing of the *FMR1* gene, resulting in a reduction or loss of fragile X mental retardation 1 protein (FMRP). FMRP is an mRNA binding protein. FMRP inhibits the translation of numerous genes involved in synaptic development and plasticity, which are essential for learning and intellectual development. The absence of FMRP causes long, thin, and immature dendritic spines, which lead to deficits in cognitive function and learning ability (1). According to the length of CGG repeats, the *FMR1* gene is divided into four types: (1) individuals with 6–44 CGG repeats are normal, and the most common sizes are 29 and 30 copies; (2) 45–54 CGG repeats are called “gray zone” or intermediate alleles; (3) premutation alleles are in the range of 55–200 CGG repeats; and (4) CGG repeats >200 are considered full mutations (2).

The full FMR1 mutation is associated with a typical FXS phenotype in males leading to mild to severe ID. Eighty-five percent of males with the FXS full mutation have intelligence quotient (IQ) scores <70, which is in the ID range. FXS in males is commonly accompanied by physical traits, such as macroorchidism, hypotonia, flat feet, soft skin, hyper-flexible joints, and distinct facial morphology, including a prominent forehead, long narrow face, and protruding ears (3). However, FXS in female individuals tends to be associated with less severe ID in which 30% have an IQ > 85 and associated behaviors include poor eye contact, anxiety, ASD, compulsive behavior, attention deficit hyperactivity disorder (ADHD), and learning disabilities (4).

Traditionally, it was believed that *FMR1* premutation carriers were not clinically involved. However, recent data have indicated medical and/or psychiatric problems associated with the carrier status. Primarily, the premutation is related to the fragile X-associated primary ovarian insufficiency (FXPOI) and fragile X-associated tremor/ataxia syndrome (FXTAS). FXPOI is defined as ovarian failure or insufficiency before the age of 40 years in women with the premutation. Approximately 20% of female premutation carriers will have FXPOI. FXTAS is a neurodegenerative disease that is observed in 40% of male and 16% of female premutation carriers. FXTAS particularly manifests in males >50 years. The core features of FXTAS include intention tremor, cerebellar ataxia, polyneuropathy, autonomic dysfunction, and cognitive decline (5). Additionally, recent studies demonstrated a higher prevalence of psychiatric and behavioral abnormalities, including hyperactivity, impulsivity, aggression, self-injury, social anxiety, and ASD, than in individuals without the premutation (6).

Size mosaicism of the CGG repeats has been observed in many patients with FXS. In the study by Nolin et al. (7), 38% of FXS patients with full mutations and mosaicism have both the premutation and full mutation alleles. The IQs of FXS patients vary vastly, ranging from normal to severe ID, based on the percentage of cells with the full mutation in the mosaicism. The probability of size mosaicism is 4/5 FXS patients; thus, the CGG size mosaicism is very common among patients with FXS (8).

## Prevalence of the *FMR1* Mutation in China

Fragile X syndrome has been extensively studied around the world, especially in Western countries. The general prevalence with a full mutation is estimated at 1/4,000 males and 1/5000–1/8,000 females (9). Since FXS is a typical sex-linked disorder, female prevalence is approximately one-half of the male rate. Moreover, males exhibit more severe impairments than females because females may have one normal X chromosome due to the random inactivation of X chromosomes in somatic cells. Premutation carriers are very prevalent in the general population; the prevalence is approximately 1 in 150–300 females and 1 in 400–850 males (6). Although FXS affects all ethnic groups, the prevalence may vary between populations. Peprah (10) showed that the incidence of FXS in countries with significant Asian populations, such as Canada, Estonia, Japan, and Taiwan was significantly lower than that in Western countries. More importantly, the prevalence of FXS in the countries that perform routine *FMR1* gene screening is higher than in countries that do not perform the test. In China, however, there have been few FXS-related studies published, due to the lack of awareness of FXS and insufficient diagnostic criteria and tools. Therefore, the epidemiologic data regarding FXS in China remains unclear.

Several previous studies have reported the prevalence of FXS in the Chinese population. Zhong et al. (11) performed a multi-institutional collaborative study utilizing molecular screening for FXS among 1,127 Chinese patients diagnosed with ID; 2.8% of patients with ID were screened for the full mutation based on the DNA analysis. The prevalence of FXS in China is equivalent to the prevalence among Caucasians, which ranges from 2.6 to 8.7% among individuals with ID. In contrast, another study performed an investigation among patients with mild ID in Hong Kong. Only 0.6% of 324 patients met the FXS diagnostic criteria. According to this result, the prevalence of FXS among Chinese individuals with ID is lower than in Western countries (12). Tzeng et al. (13) evaluated the mutant rate of the *FMR1* gene among 10,046 neonatal boys in Taiwan. The premutation and gray region mutation rate was 1:1,674 and 1:143, respectively, suggesting that the prevalence of FXS in the Taiwan was lower than in Western countries. Chen et al. (8) estimated the prevalence of FXS in mainland China. In the study, Chen et al. recruited 553 male children between the ages of 6 months and 18 years with unknown moderate to severe ID. The *FMR1* full mutation was present in 0.93% of children with unknown, moderate to severe ID. The reported FXS prevalence in this study was also lower than in Western countries. These studies indicate that a large-scale screening program would be worthwhile to determine the prevalence of FXS in the Chinese population. However, no large epidemiological studies have been conducted to date.

The reason for the different incidence of FXS among the Chinese population and Western countries may be due to several factors. First, although FXS affects all ethnic groups worldwide, the prevalence may vary among different populations because of racial differences. Second, different screening methods and varying severities of ID among the recruited populations in the various available studies may affect the reported prevalence of FXS. Additionally, most Chinese pediatricians have limited clinical knowledge of FXS and the associated complex comorbidities, which may also affect the assessment of the disease. Very little basic and clinical research on FXS occurs in China compared to Western countries that lead the world in the FXS research (9). Moreover, in younger patients, the typical physical characteristics of FXS, such as a long narrow face, protruding ears, and macroorchidism, are less obvious (becoming more apparent as the child approaches adolescence). This lack of early physical characteristics increases the clinical diagnostic challenge regarding FXS for most Chinese pediatricians. To date, confirming an FXS diagnosis depends on the detection of *FMR1* mutations through molecular genetics testing, particularly in younger children. However, not many hospitals and institutions can perform genetic testing for *FMR1* mutations in China. Therefore, many FXS patients may have gone undiagnosed due to the reliance on clinical diagnoses, particularly in younger children. The low diagnostic rate of FXS further results in limited awareness of FXS among the public and medical professions and creates a positive feedback loop. Additionally, ASD and FXS are not currently included in routine medical school training in China. Li et al. (14) performed a study on FXS knowledge and attitudes toward FXS among Chinese medical college students. Approximately only 30% of these students have heard of FXS but are not very familiar with FXS. This study highlights the need to increase awareness of FXS in China.

## Targeted Treatment for FXS

Fragile X mental retardation 1 protein, the translation product of *FMR1*, is a translational regulation factor that can control most of the proteins important for synaptic maturation and plasticity. The absence of FMRP results in significant alterations in learning and cognition and abnormal synaptic plasticity, including immature, elongated spines, and enhanced long-term depression (LTD) (meaning the weakening of synaptic connections). Overall, the degree of FMRP deficiency is positively correlated with the severity of FXS and intellectual impairment (15). Furthermore, we have gained much understanding regarding the neurobiology and mechanisms of FXS from studies of the *FMR1* knockout (KO) mouse. Based on evidence from the *FMR1* KO mouse model and early preliminary clinical studies, abnormal synaptic plasticity in FXS can be rescued by targeted treatments.

The metabotropic glutamate receptor 5 (mGluR5) pathway is one of the pathways dramatically affected by the absence of FMRP. The absence or loss of FMRP will lead to enhanced mGluR5 activity, which results in LTD or abnormal synaptic connections (16). Thus, treatment of FXS patients with mGluR5 antagonists may improve LTD and the abnormal synaptic plasticity associated with FXS. *FMR1* KO mice models have shown that mGluR5 antagonists can reverse the social manifestations and behaviors of FXS. Thus, the mGluR5 signaling pathway has great significance for social interactions. mGluR5 antagonists may serve as potential therapeutic drugs to alleviate the behavioral problems exhibited by FXS patients (17). In the acute phase, hippocampal LTD and protein synthesis can be reversed by treatment with mGluR5 antagonists in *FMR1* KO mice models. In the chronic treatment phase, mGluR5 antagonists can improve cognitive ability, auditory hypersensitivity, and abnormal synaptic plasticity. Additionally, mGluR5 antagonists are helpful for treatment of macroorchidism. Also, younger children respond better to drug intervention than adults (17, 18). These findings have provided a basis for the development of mGluR5 antagonists, such as mavoglurant (AFQ056), RG7090, and fenobam, to be used in human clinical studies.

A multicenter randomized, double-blind study evaluated the efficacy and safety of mavoglurant, an mGluR5 antagonist, in adolescent patients with FXS. The efficacy of mavoglurant was not confirmed in FXS patients (19). Bailey et al. (3) also used these data to show that FXS patients treated with mavoglurant did not exhibit a better treatment response than FXS patients receiving placebo as measured by the Clinical Global Impression-Improvement (CGI-I). Previously, Jacquemont et al. (20) performed a randomized, double-blind, crossover study to examine whether AFQ056 could improve the behavioral symptoms of FXS patients. Thirty male patients with FXS aged 18–35 years were included. According to the primary outcome measure, the Aberrant Behavior Checklist-Community Edition (ABC-C) score, no significant effects were found at day 19 or 20 following AFQ056 treatment. However, when an exploratory analysis was performed also measured by the ABC-C score, some of the FXS patients who received AFQ056 treatment exhibited significant improvements compared to those receiving placebo. However, the study by Berry-Kravis et al. (19) did not show a difference between patients with a full mutation and mosaic individuals. Earlier, Berry-Kravis et al. (21) conducted a trial with fenobam, also an mGluR5 antagonist, in 12 adults (6 males and 6 females) with FXS. There are 9 of 12 (5 males and 4 females) FXS patients receiving fenobam exhibited improvements in eye contact, social ability, anxiety, and motor overactivity compared to those receiving placebo. Most importantly, no significant side effects were observed in the fenobam treatment group. Particularly, no central nervous system adverse events were associated with fenobam treatment. The mGluR5 antagonist is relatively safe for individuals with FXS. Although mGluR5 antagonists were not significantly helpful in most studies of adolescents and adults, a large multicenter study using mavoglurant in children 3–6 years old with FXS combined with intensive language intervention will take place (funded by NeuroNext) in the United States during the next few years. It is likely that mavoglurant will be more efficacious in young children than older individuals with FXS.

In addition to the mGluR5 pathway, the GABA system is another dysfunctional neurotransmitter system in FXS patients. GABA is an important inhibitory neurotransmitter in the brain. The balance between Glu and GABA is essential for human learning and memory. Some studies demonstrated that the GABA system is downregulated in animal models of FXS. There are several GABAergic synapse components that change expression in the *FMR1* KO mouse model. Additionally, crucial synaptic proteins were reduced, including GABA_A_ receptors and enzymes involved in GABA production and metabolism. These findings have prompted many clinical trials of GABAergic drugs for FXS (22, 23). A phase II study of STX209 (arbaclofen), a GABA_B_ agonist, was conducted in 63 children and adults with FXS, and the Aberrant Behavior Checklist-Irritability subscale was the primary outcome measure. No significant difference was detected between those receiving STX209 and those receiving placebo. In the subsequent exploratory study, STX209 also did not yield a better result than placebo in FXS patients. However, the visual analog scale (VAS), which was used to rate parent-nominated problem behaviors, indicated significant improvement in the STX209 group. A *post hoc* analysis also revealed a significant benefit of STX209 treatment based on the ABC-Social Avoidance scale. STX209 was well tolerated, and the most common side effects were sedation and headache, which occurred in 8% of FXS patients receiving STX209 treatment. Thus, although GABA_B_ agonists were not significantly helpful for patients with FXS, they may have the potential to improve social function and behavior and are well tolerated by patients with FXS (24).

A reduction or loss of FMRP in FXS is also associated with the expression of matrix metalloproteinase 9 (MMP9). MMP9 plays an important role in hippocampal synaptic maturation and plasticity. Bilousova et al. (25) found that hippocampal MMP9 levels were elevated in the *FMR1* KO mouse. The authors also evaluated treatment with minocycline, a tetracycline analog, in *FMR1* KO mice. MMP9 levels were lowered after minocycline treatment. Minocycline can improve abnormal synaptic plasticity and improve behavior in *FMR1* KO mice. Leigh et al. (26) conducted a controlled trial to confirm the efficacy of minocycline in 55 FXS patients ranging from 3.5 to 16 years old. As measured by the CGI-I, minocycline treatment produced significant improvements compared to placebo. Additionally, anxiety and mood-related behaviors in the minocycline group were greatly reduced compared to those in the control group (VAS scores).

Additionally, treatment of FXS-related psychiatric and physical problems is very important. One important characteristic of FXS is the high rate of comorbid seizures, especially in FXS patients diagnosed with ASD. The co-occurrence of seizures is about 14% in males and 5% in females with full *FMR1* mutations. Seizures in FXS are more often focal. The high-risk age for onset occurs from 4 to 10 years. In addition, these patients, including 77% with seizures and 23% without seizures, showed an epileptiform abnormality in EEG findings. And they may share some benign epilepsy features with centrotemporal spikes. A total of 88% (14/16) with seizures are easily to control with one or two antiepileptic drugs (27). However, although seizures in FXS patients are usually easy to control, they are also associated with the overall severity of FXS, particularly regarding the comorbidities, which include ASD and anxiety. Seizures impact the prognosis and quality of life of patients with FXS. Active antiepileptic therapy for FXS and seizures is necessary (28). Furthermore, stimulants, selective serotonin reuptake inhibitors, and antipsychotic medications are used for the treatment of ASD, anxiety, impulsive behavior, ADHD, or aggressive behavior (29). A recent study demonstrated that a low dose of sertraline (2.5–5.0 mg/day) for a 6-month period was beneficial for overall development, visual reception, and fine motor coordination in young children with FXS aged 2–6 years (30). The young children with FXS plus ASD also demonstrated significant improvements in expressive language abilities as measured by the Mullen Scales of Early Learning (30). These early treatment data demonstrate the importance of early diagnosis of FXS so that interventions can be started early.

Although most studies have focused on drug treatment for FXS, several non-pharmacological interventions can be used to treat the condition. Their purpose is to improve behavioral problems of FXS patients, such as attention difficulties, social anxiety, poor eye contact, stereotypic, or repetitive behaviors. Early intensive behavioral interventions are able to stimulate the development of social communication and language, joint attention, and daily living skills. Applied behavior analysis (ABA), based on functional analysis, can be used to find out the environmental factors that lead to behavioral problems, and through training to improve the behavioral problems of children with FXS (31). Treatment and education of autistic and related communication-handicapped children, floor time, and relationship development intervention are also used to treat behavioral problems of FXS (32). These supportive interventions significantly impact the academic outcome and quality of life and are extremely important for the life of these children and their families. Another study carried out a trial of Cogmed working memory training in FXS. The evidence shows that Cogmed training can effectively improve the working memory ability of FXS and reduce the symptoms of ADHD (33). Therefore, behavioral therapy may have a synergistic effect with pharmaceutical targeted treatments that will further improve the cognitive ability and behavioral problems of FXS. Importantly, the behavioral interventions for autistic like features are becoming more widely used in China.

However, to date, there are no guidelines for the diagnosis and management of FXS in China. Moreover, no targeted drug has been used to the Chinese market so far. Most of the treatment of FXS patients mainly relies on symptomatic treatment of ASD, ADHD, or seizures. The biggest obstacle is the limited awareness about FXS among medical professionals, the public, and relevant departments. Thus, although China has a potentially large number of FXS patients, basic and clinical trials for FXS patients have not been conducted extensively in China. Additionally, there is a relatively weak basic and clinical research foundation for FXS in China compared with Western countries. Currently, nationwide healthcare programs in China cover many basic general health-care expenses. For example, neonatal screening has developed rapidly in recent years, including for phenylketonuria and congenital hypothyroidism (34). Furthermore, general screening for Down syndrome also has been conducted as part of routine prenatal testing in China. However, no national healthcare plans cover genetic testing for FXS. Medical insurance does not cover the high cost of FXS screening. Thus, many families in underdeveloped rural areas cannot afford the cost. This may delay treatment for FXS beyond the optimal time. Even after diagnosis, behavioral problems in children with FXS often require lifelong support, which presents a significant burden for families. Working with individuals with FXS will need the combined efforts of the family, physicians, and educational system to provide behavioral training and other management techniques. The safety and tolerability of therapies for FXS patients are also important concerns for Chinese families. To conduct treatment for FXS patients in China, it is important to obtain the support of the government and health-care sectors and attract public attention by providing information on the importance of active treatment for FXS. Population safety and tolerability of treatment modalities should also be evaluated. Due to the above reasons, the long-term quality of life in FXS is not optimistic in China. A study, which evaluate the outcome of Chinese patients with FXS by using social adaptive behavior to represent the quality of life, find that the adaptive behavior of children with ID is closely related to the quality of life. Compared with Down syndrome and typically developing boys, boys with FXS had significantly lower scores in the work skills, socialization and self-management, after age and receptive language ability matched. The findings suggest that patients with FXS have much more difficulties in the life living than the other two groups. Furthermore, the level of language comprehension of Chinese FXS patients was significantly lower than that of the patients in western developed countries (35). Therefore, there is a long way to go toward the diagnosis and treatment of FXS in China.

Recently, an increasing number of clinicians, researchers, and the government in China are becoming aware of the significant hazards and economic burdens of rare diseases, especially chronic diseases. Thus, the prevention and treatment of rare diseases, including FXS, has gained increasing attention. We have also begun to realize the importance of resource allocation and basic and clinical research in China. At the moment, projects on rare diseases are being implemented nationwide in China. The purpose of this promotion is to increase the study of rare diseases in China and to be in line with the world in the field of FXS (36). Although we have begun to improve in the field of FXS, substantial gaps still exist between China and Western developed countries. Accordingly, we should learn advanced Western technology and research methods to make progress in the testing and treatment of FXS. Additionally, coupled with the increased awareness of public and government and a significant amount of investment, China has the potential to make outstanding contributions to the diagnosis and treatment of rare diseases, including FXS.

## Genetic Counseling to Prevent FXS

Although CGG repeats are stable when they are in the normal range, they show genetic instability in the gray mutation or premutation regions. The premutation frequently expands to the full mutation with maternal transmission. As the length of CGG repeats increases, the risk of amplification increases. Additionally, it is influenced by the number of AGG interruptions. The absence of AGG interruptions will lead to more instability of the *FMR1* gene (7). A study showed that more than 94% of *FMR1* alleles with >90 CGG repeats will expand to full mutations, and 56 CGG repeats is the smallest number of repeats that can be extended to a full mutation in one generation (7). In general, there are one or more AGG interruptions inserted into the CGG repeats. These interruptions may influence *FMR1* allele stability by stabilizing strand slippage during DNA replication. The AGG interruptions among the CGG repeats increase the stability of the *FMR1* allele and decrease the risk of expansion to full mutations during maternal transmission. Because of the effect of AGG interruptions on *FMR1* allele stability in gray mutation or premutation regions, they should be considered when conducting genetic counseling (37).

Nolin et al. (37) evaluated the effect of AGG interruptions and CGG repeats on *FMR1* allele stability in the gray region and found small premutation alleles with 45–69 CGG repeats. Both the number of AGG interruptions and the length of CGG repeats were associated with allele instability during maternal transmission. Maternal alleles with no AGG interruptions have the highest risk of expansion to full mutation during transmission. All of the full mutation expansions in this study were from mothers with no AGG interruptions in the CGG repeats. The results suggest that the AGG interruptions can reduce the stability of gray region and small premutation alleles with 45–69 CGG repeats during transmission. Therefore, combined consideration of the number of AGG interruptions and the length of CGG repeats during genetic counseling will provide a more accurate risk assessment of expansion to full mutations. Recently, another study examined the stability of maternal and paternal alleles with 45–90 repeats during transmission and assessed the effect of AGG interruptions on CGG repeat instability. Approximately, half of the full mutation expansions occurred in mothers with no AGG interruptions, while 43% of the full mutation expansions occurred with one AGG interruptions. Only 4% of full mutation expansions resulted from mothers with two AGG interruptions (7).

Although the length of CGG repeats and the number of AGG interruptions are significant factors influencing *FMR1* allele stability in the gray region and premutation alleles on transmission, other factors, such as maternal age, are likely to have a role when considering the risk of expansion to full mutation. Yrigollen et al. (38) found that the length of CGG repeats, the number of AGG interruptions, and maternal age are the most important factors when considering the risk of premutation expansion to full mutations during maternal transmission. Interestingly, maternal age was related to the risk of expansion to full mutation during maternal transmission. The risk of expansion to full mutation increases with age. For example, the probability of expansion to full mutation is 56% for a 20-year-old mother. However, the risk increases to 85% when the mother is aged 30 years. This suggests an additive effect of maternal age and allele instability. In addition to the effects of the length of the CGG repeat and the number of AGG interruptions on stability of *FMR1* alleles, maternal age is also a significant factor when considering the risk of expansion to a full mutation. That is, a younger mother may have a lower risk of having expansion to full mutation in a child with CGG repeats. However, in the study by Nolin et al. (7), the effect of maternal age on the risk of expansion to full mutation revealed only a possible association with transmission (no significant difference). The reason may be that maternal age has less impact on *FMR1* allele stability than CGG repeats and AGG interruptions when considering the risk of expansion to full mutation. Therefore, more evidence is needed to further demonstrate the effect of maternal age on the amplification risk of *FMR1* alleles.

The accurate calculated risk rate of expansion to full mutation CGG repeats will be an important information for the genetic counseling of families with individuals with FXS and premutation carriers who want to have children. A calculation model that includes the length of the CGG repeat, the number of AGG interruptions, and maternal age is more precise for genetic counseling. Women who carry CGG repeats in premutation regions with no AGG interruptions have a risk of expansion to full mutation. Thus, these women should receive prenatal genetic counseling. Although maternal alleles with one or two AGG interruptions have lower risks than those with no AGG interruptions, they still have a risk of amplification to full mutation according to the number of CGG repeats. If a premutation carrier can be identified in time and the appropriate measures taken, FXS can be reduced or prevented in maternal transmission to some degree. Broad screening of women during early pregnancy or among those who wish to become pregnant is considered a good approach to identify carriers with significant risk of expansion to full mutation during maternal transmission. However, whether to accept the FXS prenatal screening should be a personal decision. Therefore, improved public awareness of FXS during early prenatal genetic screening can greatly reduce births of FXS children.

Obviously, Down syndrome is familiar to Chinese people. However, not everyone recognizes FXS. Although genetic counseling has become very advanced in Western countries, only a few hospitals and institutions can perform genetic testing for FXS in China. The main reason is that the number of CGG repeats cannot be evaluated accurately. Molecular diagnostic tests for FXS include region-specific CGG PCR amplification and Southern blot analysis. Recently, although, some commercial FXS testing trial kits have been introduced in China, the high price and technical constraints have hindered widespread use of the kits for genetic testing and prenatal diagnosis. Moreover, until now, the cost of genetic testing for FXS has not been covered by medical insurance in China as is screening for congenital hypothyroidism and phenylketonuria (9). The cost of genetic testing for FXS is a significant family expense, especially in underdeveloped areas. Therefore, many of FXS are underdiagnosed or misdiagnosed in clinic. At present, there is relatively scarce evidence on FXS in China, which is partly due to lack of awareness of FXS among doctors, the public, and the government. A wide range of actively screening for FXS children is rare in China. Unless a highly clinical suspicion, pediatricians will think of this condition. It may lead to more and more FXS offspring patients in China and will make management and treatment more difficult. In addition, treatments are not always administered in the Chinese FXS population. Most importantly, FXS treatment is a long-term supportive treatment. Once diagnosed, further medical treatment will be a substantial financial burden for a family. FXS management also brings great mental stress to the family and society. Therefore, it is critical to consider the basic national condition of China, which is still a developing country with lagging economic strength compared to developed Western countries. Moreover, China has a very large population. The unique characteristics of China require us to provide appropriate methods for the diagnosis and treatment of FXS. First, we must improve FXS awareness among doctors, the public, and the government. Second, special guidelines should be created by Chinese language experts to guide doctors how best to describe aspects of FXS to patients. Moreover, in the current era of network information, we should make full use of network resources to introduce basic knowledge of FXS to the Chinese public (14).

The prevention and treatment of FXS will be the result of comprehensive efforts in various arenas, including government, medical personnel, and the public. A specialized clinic and research center is an urgent necessity in order to provide the latest knowledge to Chinese medical professionals. More publicity and education are needed to provide information and help to individuals with FXS and their families.

## Author Contributions

MN contributed to conception and design, drafted the manuscript, critically revised the manuscript, and made final approval of the version to be published; YH contributed to conception and design, critically revised manuscript, made final approval of the version to be published, and agreement to be accountable for all aspects of the work; AD contributed to conception and design, critically revised the manuscript, and made final approval of the version to be published; JD contributed to conception and design and made final approval of the version to be published; HJ, QL, and RH contributed to conception and design, critically revised manuscript, and made final approval of the version to be published; JQ contributed to conception and made final approval of the version to be published; JZ contributed to conception, critically revised manuscript, and made final approval of the version to be published.

## Conflict of Interest Statement

The authors declare that the research was conducted in the absence of any commercial or financial relationships that could be construed as a potential conflict of interest.
